# Releasable Suture versus Autologous Blood for Pterygium Surgery using Conjunctival Autografts

**DOI:** 10.18502/jovr.v15i1.5938

**Published:** 2020-02-02

**Authors:** Gautam Singh Parmar, Bhushan Ghodke, Ashok Kumar Meena

**Affiliations:** ^1^Department of Cornea and Refractive Services, Sadguru Seva Sang Trust, Chitrakoot, Madhya Pradesh, India

**Keywords:** Autologous Blood, Conjunctival Autograft, Pterygium, Recurrence, Releasable Suture

## Abstract

**Purpose:**

To evaluate the efficacy of releasable single suture (RS) for conjunctival autograft (CAG) and to compare it with sutureless gluefree (SG) technique in pterygium surgery.

**Methods:**

We conducted a retrospective comparative study on patients with primary pterygium who underwent CAG. In 150 patients, CAG was additionally secured by a single 10-0 nylon releasable suture (RS) which was released on the first postoperative day. In 47 patients, no suture was applied, and CAG was allowed to stick to the scleral bed by autologous fibrin only (SG group). The duration of surgery and size of CAG (in mm${}^{2}$) was noted in both groups. All patients completed one year of follow-up. Factors that were studied included graft stability, patient comfort, complications, and recurrence.

**Results:**

The mean age of patients in RS and SG groups was 39.6 ± 11.8 and 47.3 ± 13.8 years, respectively. The mean duration of surgery was 4.84 ± 1.34 min in RS group and 4.90 ± 1.42 min in SG group (*P* = 0.001). The size of CAG used in both groups was comparable with more stability in RS group postoperatively. Graft retraction rate in RS group was 5.3% (1 mm retraction in CAG more than 36 mm${}^{2}$) with no event of graft loss. The graft loss occurred in 6.3% of eyes in SG group. The recurrence rate in RS group was 4%, while in SG group it was 6.3% (*P* = 0.4).

**Conclusion:**

RS, by augmenting the autologous blood mechanism, may offer an easy to learn option for pterygium surgery with good stability even in large sized CAGs.

##  INTRODUCTION 

Pterygium is a triangular “wing-like” growth consisting of conjunctival epithelium and hypertrophied subconjunctival connective tissue that encroaches onto the cornea.^[1]^ The surgical excision is the treatment of choice, but its propensity for recurrence is a major concern. Switching from unacceptably high recurrence rate of bare sclera technique (60–80%)^[2]^ to encouraging results of conjunctival autograft (CAG) was popularized by Kenyon et al.^[3]^ To date, CAG remains a time-tested and gold standard treatment with low recurrence and high stability.^[3,5]^ But, the debate over the best approach is focused on whether surgeons should use sutures or suture-less options like fibrin glue or autologous fibrin to secure the CAG.

Fibrin glue has shorter duration of surgery, better patient comfort, and less chance of recurrences (14%) compared to other techniques for CAG fixation.^[4]^ Important disadvantages are cost and accessibility. In fibrin-assisted surgery, complications such as dehiscence, graft retraction, and pyogenic granuloma were seen more frequently.^[5,6]^

Suture assisted surgery ensures graft stability with acceptable recurrence rates of 5–15%.^[7]^ The immediate postoperative patient discomfort with symptoms of pain, watering, foreign body sensation, and sub-conjunctival hematoma are its major disadvantages.^[8,9]^

Thus, we proposed a newer suture assisted technique in which the CAG was secured by a single 10-0 nylon suture with a releasable knot and was released on the first postoperative day. We compared its results with current accepted standard of autologous blood assisted pterygium surgery.

##  METHODS

We conducted a retrospective comparative observational study at our tertiary eye care center. Medical records of 197 patients with primary pterygium were reviewed. All methods adhered to the tenets of The Declaration of Helsinki Principles for research in human subjects. The treatment groups were assigned arbitrarily by one of the authors. In the RS group (*n* = 150), CAG was secured by a single 10-0 nylon suture tied with a releasable knot. In the SG group (*n* = 47), patients' own blood from the surgical area was used as a source of fibrin. We followed the slit-lamp grading system given by Tan et al.^[10]^ All patients were operated by a single surgeon.

Primary outcome measures were graft stability and recurrence. Secondary outcome measures were patient comfort in terms of pain on Visual Analogue Scale (VAS) and best corrected visual acuity (BCVA).

Descriptive and inferential statistical analysis was carried out in the present study. Chi-square/Fisher Exact test was used to find the significance of study parameters on categorical scale between the two groups. The significance was assessed at 5% level of significance. The statistical software namely SPSS 15.0 was used for the analysis of the data and Microsoft Word and Excel were used to generate graphs and tables.

##  Surgical Technique

**Figure 1:**
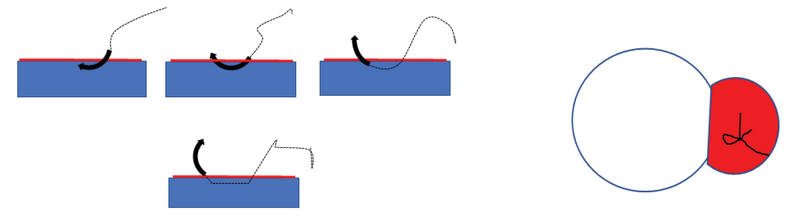
Diagrammatic representation of releasable single suture in situ.

**Figure 2:**
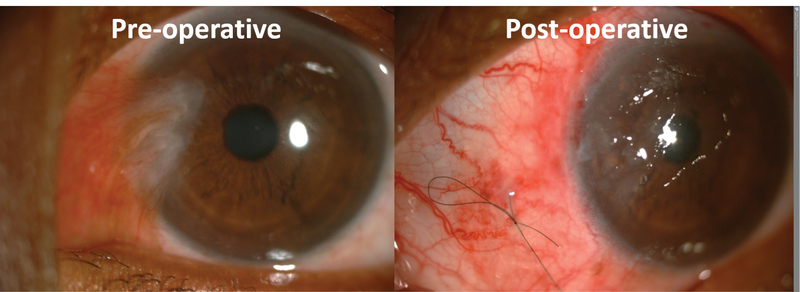
Preoperative and postoperative slit-lamp photograph of patient 1.

The body of pterygium was firmly held at limbus by toothed forceps, and adhesion between the pterygium and sclera was sharply incised and separated using conjunctival scissors. The head of pterygium was avulsed from the underlying cornea. The fibrous tissue was scrapped off by Bard-Parker 15 number blade. Only the fleshy subconjunctival hypertrophied tissue with tortuous vessels and adjacent thickened conjunctival tissue was excised. The size of the bare-scleral defect was measured in millimeter square (mm${}^{2}$) by Castroviejo calipers. CAG was taken from superior quadrant by ballooning the conjunctiva with 0.5 cc dexamethasone (4mg/ml) subconjunctival injection. A thin tenon-free CAG was prepared. Graft flip was done with an iris spatula and was transferred to bare sclera carefully to coincide the limbal to limbal area and keeping the epithelial side up. Ironing of the graft was done with two iris spatulas. In RS group, though the CAG was already stuck to the underlying bare sclera due to autologous fibrin, the center of CAG was additionally stabilized (see Figure 1, showing diagrammatic representation of a releasable suture (RS) in situ) by single 10-0 nylon suture tied in a releasable fashion keeping one end of suture longer than the other (see video, electronic supplementary material 1, showing the application of RS). In SG group, bleeding secondary to excision of vessel-rich tissue was used as a source of autologous fibrin. Excessive bleeding was controlled by hemostasis with the help of sterile Merocel sponge to avoid graft relift. Graft was examined for apposition. End of surgery was noted after the removal of the eye speculum. After 24 h, on the first postoperative day, patient was examined using a slit-lamp (see Figure 2, showing preoperative and postoperative slit-lamp photograph of patient 1). Proparacaine 0.5% eye drop was used as topical anesthetic agent and the suture was released at slit-lamp by pulling the longer end of the 10-0 nylon suture in RS group; patient was re-examined for graft status, apposition, and retraction. The VAS with grading from 1 to 10 was shown and explained to patients, asking them to report the severity of pain after the release of suture on the first postoperative day. Postoperatively, all patients were given low potency steroid eye drops four times a day for the first week and tapered off over six weeks, while antibiotic drops were administered four times a day for two weeks. All patients were examined at week 2 and at months 3, 6, and 12. During each visit, patients were evaluated for visual acuity, graft apposition, pain on VAS, complications, and pterygium recurrence. Recurrence was defined as any fibrovascular regrowth extending beyond the surgical limbus involving the cornea.

##  RESULTS 

Both groups were comparable regarding the demographic profile [Table 1]. The mean age of patients was 39.6 ± 11.8 years and 47.3 ± 13.8 years in RS and SG groups, respectively. The primary pterygium was present on the right side in the majority of patients. The predominant type of pterygium in our study was of Tan's grade 2 (60% in RS group and 68% in SG group); while 40% of patients were of Tan's grade 1 in RS group; and 29% of patients were of Tan's grade 3 in SG group. The mean duration of surgery in RS group was 4.84 ± 1.34 min with minimum of 3 min and maximum of 10 min. In SG group, the mean duration of surgery was 4.90 ± 1.42 min. In RS group, 36% of patients complained of pain of scale 2 following the release of suture on the first postoperative day, while in SG group, 44% of patients had a pain scale of grade 2. Mean BCVA before surgery was 0.27 ± 0.41 and 0.22 ± 0.33 LogMAR which improved to 0.16 ± 0.28 and 0.16 ± 0.22 LogMAR at 2 weeks after surgery and was maintained thereafter till the last follow-up visit in RS and SG groups, respectively. The maximum area of CAG was 42mm${}^{2}$ in both RS and SG groups. In SG group, one patient suffered from the event of graft loss because of eye rubbing. A second procedure was done on the same day and a second CAG was derived from the contralateral eye after obtaining an informed consent from the patient. While in RS group, eight (5.3%) patients in whom the CAG was greater than 36mm${}^{2}$, the graft was retracted by 1 mm from the nasal margin during the release of the suture on the first postoperative day, which was successfully repositioned by graft ironing. In seven (4.6%) patients in the RS group, there was failed attempt in releasing the suture. In these patients, the suture was then cut with Vanna's scissor and removed. In six (4 %) patients in the RS group and three (6.3%) patients in the SG group, there was a recurrence of grade 1 (conjunctival), which did not involve the cornea at one year postoperatively. No patients were lost to follow-up.

**Table 1:** The details of demographic and other variables in respective groups


**VARIABLES**	**CATEGORIES**	**RS GROUP (** ***n*** ** = 150) (RS-Releasable Suture)**	**SG GROUP (** ***n*** ** = 47) (SG-Sutureless Gluefree)**	**** ***P*** **-value**
**AGE (in years)**	< 20	1(0.7%)	0(0%)	
	20–30	42(28%)	8(17%)	
	31–40	51(34%)	11(23.4%)	
	41–50	36(24%)	9(19.1%)	
	51–60	12(8%)	11(23.4%)	
	> 60	8(5.3%)	8(17%)	
	**Mean age (years)**	**39.65 ± 11.85**	**47.36 ± 13.84**	
**GENDER **	Male	62(41.3%)	25(53.2%)	0.15
	Female	88(58.7%)	22(46.8%)		
**EYE INVOLVED**	Left	51(34%)	22(46.8%)	0.11
	Right	99(66%)	25(53.2%)		
**PTERYGIUM GRADE**	1	60(40%)	1(2.1%)	0.54
**(TAN et al)**	2	90(60%)	32(68.1%)		
	3	0	14(29.8%)		
**DURATION OF SURGERY**	1-5	109(72.7%)	35(74.4%)	0.86
	6-10	41(27.3%)	12(25.5%)		
	**Mean duration (minutes)**	**4.84 ± 1.34**	**4.90 ± 1.42**	
**RECURRENCE**	6 (4%)	3 (6.3%)	0.495

##  DISCUSSION

From bare sclera technique to mini-simple limbal epithelial transplant,^[11]^ surgical techniques for pterygium are constantly evolving. The majority of these techniques require sutures with postoperative suture related complications.^[12,13]^

This retrospective comparative study showed no significant difference in recurrence rate of pterygium after surgery in RS and SG groups. Both techniques were equally effective in lowering the recurrence rate after pterygium surgery. However, with addition of a simple step of applying a single RS to the autologous fibrin technique, the surgeon can be assured of not losing the CAG completely in cases with graft loss. This is important in patients coming from low socio-economic strata in developing countries, where the post operative period is complicated by a number of factors, especially eye rubbing.

Compared to sutures, sutureless options such as fibrin glue, introduced by Koranyi et al,^[4]^ and autologous blood^[14,15]^ are more popular adjuvants for CAG. Although considered safe, the theoretical potential risk of transmission of infections like parvovirus-B19, hepatitis B virus, human immune-deficiency virus, and anaphylaxis in susceptible individuals make fibrin glue, an “off-label” adjuvant by Food and Drug Administration (FDA) for the use in ophthalmology.^[14,16]^ On the other hand, the cost of fibrin glue is equal to five sutures making it less cost-effective.^[16]^ Improper adherence of fibrin glue causing graft loss is a recognized complication.^[17,18]^ In last few years, several studies published results of autologous blood as a biological agent in pterygium surgery. Commercially available fibrin glue consists of two components – sealer protein solution (human fibrinogen and a synthetic fibrinolysis inhibitor, aprotinin) and the thrombin solution (human thrombin). When mixed together, these two components combine and mimic the final stages of natural clotting cascade to form a cross-linked fibrin clot. Whereas, the mechanism of action of autologous blood as a sealant is the natural clotting cascade resulting in fibrin polymerization. Autologous fibrin is associated with disadvantages such as graft dehiscence and graft loss. In these patients with graft loss, a second surgery is generally required in which the CAG is retrieved from the fellow eye being ultimately and reliably affixed by sutures.^[19]^ In our study, there was one patient in SG group who suffered graft loss on the first postoperative day due to eye rubbing. The CAG was then retrieved from the contralateral eye after proper counselling and written consent. In contrast, no event of graft loss occurred in the RS group. The most important step in patients operated with RS was releasing the suture on the first postoperative day. We experienced graft retraction of 1 mm in eight (5.3%) patients with large grafts (more than 36 mm${}^{2}$), after the suture was released. Also, pulling the long end of the RS posed some difficulty in seven (4.6%) patients, which was then managed with Vanna's scissor.

In RS group, the suture helped in proper and uniform adherence of autograft to the scleral bed during the immediate postoperative period especially first 24 h. The mean surgical time in the RS group was 4.84 ± 1.34 min, while a relatively longer mean operating time of 4.90 ± 1.42 min in the SG group was present which was statistically insignificant (P=0.86). Studies in the past demonstrated the correlation of surgical time and success of pterygium surgery.^[14]^ More intraoperative time leads to increased postoperative reaction and leads to infection.^[20]^ The short time taken to apply a single RS means more pterygium surgeries in less time.

The pain scale on VAS was consistently less than 2 in all 197 patients on the first postoperative day. This result is comparable to studies in which fibrin glue^[4]^ or autologous blood^[14,16]^ is used. Complications like serious inflammatory reaction, infection, corneal ulceration, scleral melting or dellen were not noticed in any of the patients.

Mitra et al^[20]^ mentioned that once the CAG stays in place for the first 24–48 h, it is going to stick around. Thus, the use of single suture for the first 24 h, as a simple additional step to the autologous fibrin technique, gives a psychological relief to the operating surgeon. Hirst et al^[21]^ reported that 97% of pterygium recurrences develop within the first 12 months of surgery. In our study, the conjunctival recurrence rate was 4% in the RS group and 6.3% in the SG group, which is comparable to the reports from other similar studies.

There are several limitations in our study. Firstly, the retrospective study design has its inherent limitation. Secondly, a relatively small sample size of 197 patients. Third, a follow-up period of one year may not be sufficient to comment on the efficacy of our technique with respect to the recurrence. Further evaluation is required to study the recurrence rate and complications after RS, with larger sample size and randomized controlled trials. We recommend that the length of RS over the graft surface should be adequately long as governed by the area of CAG. Further studies are required to find the correlation of suture length and the area of CAG and its impact on the graft stability.

In conclusion, in pterygium surgery, RS did not significantly reduce postoperative pterygium recurrence. However, in cases with large-sized CAGs and in patients with increased frequency of eye rubbing, RS may offer an easy-to-learn and reliable method by augmenting the autologous fibrin mechanism. The postoperative patient comfort is comparable to the currently accepted standard of sutureless gluefree (SG) technique for pterygium surgery.

##  Supplemental video

Video showing technique of application of the releasable suture.
